# EXPLORING COMMUNICATION DISPARITIES IN LATE OLD AGE THROUGH THE LENS OF COMMUNICATION INFRASTRUCTURE THEORY

**DOI:** 10.1093/geroni/igac059.2195

**Published:** 2022-12-20

**Authors:** Carrie Leach, Thomas Jankowski

**Affiliations:** Wayne State University, Detroit, Michigan, United States; Wayne State University, Detroit, Michigan, United States

## Abstract

Health disparities are driven, in part, by communication disparities. Those who face the greatest health burdens often have the least communication resources to draw on to help them to manage and cope. As part of a multi-year, multi-level community engaged study to understand how to reduce the communication disjuncture between community-based service providers and older adults age 75 and older, we draw on data collected from 1,609 older adults through in-depth interviews (n=20) and a random sample survey (n=989) in one county in Michigan, along with a random digit dial telephone survey (n=600) conducted statewide. Findings point to key channels for disseminating information to hard-to-reach older adults being healthcare professionals and home delivered mail. Despite optimistic reports that technology usage and adoption rates are increasing for older users, findings suggest that old adults continue to face digital exclusion. Challenges with connecting through interpersonal networks are discussed. Limited resources for connecting with others through technology combined with increased social distancing due to the pandemic may exacerbate older adults’ diminishing social networks and dwindling communication resources to draw upon for managing their health in later old age. Understanding how to connect with the hard-to-reach is pressing particularly as connecting via tele-health normalizes. This information may be employed to inform strategies for disseminating information to older adults, including those ages 75 and older who are hardest-to-reach, and likewise, outreach and recruitment by researchers to engage them in studies that aim to reduce health disparities.

